# MicroRNA-mediated gene regulation plays a minor role in the transcriptomic plasticity of cold-acclimated Zebrafish brain tissue

**DOI:** 10.1186/1471-2164-12-605

**Published:** 2011-12-14

**Authors:** Ruolin Yang, Zhonghua Dai, Shue Chen, Liangbiao Chen

**Affiliations:** 1 State Key Laboratory of Molecular Developmental Biology, Institute of Genetics and Developmental Biology, Chinese Academy of Sciences, Beijing 100190, China; 2 Graduate School of the Chinese Academy of Sciences, Beijing 100080, China

## Abstract

**Background:**

MicroRNAs (miRNAs) play important roles in regulating the expression of protein-coding genes by directing the degradation and/or repression of the translation of gene transcripts. Growing evidence shows that miRNAs are indispensable player in organismal development with its regulatory role in the growth and differentiation of cell lineages. However, the roles of miRNA-mediated regulation in environmental adaptation of organisms are largely unknown. To examine this potential regulatory capability, we characterized microRNAomes from the brain of zebrafish raised under normal (28°C) and cold-acclimated (10°C, 10 days) conditions using Solexa sequencing. We then examined the expression pattern of the protein-coding genes under these two conditions with Affymetrix Zebrafish Genome Array profiling. The potential roles of the microRNAome in the transcriptomic cold regulation in the zebrafish brain were investigated by various statistical analyses.

**Results:**

Among the total 214 unique, mature zebrafish miRNAs deposited on the miRBase website (release 16), 175 were recovered in this study. In addition, we identified 399 novel, mature miRNAs using multiple miRNA prediction methods. We defined a set of 25 miRNAs differentially expressed under the cold and normal conditions and predicted the molecular functions and biological processes that they involve through Gene Ontology (GO) annotation of their target genes. On the other hand, microarray analysis showed that genes related to mRNA processing and response to stress were overrepresented among the up-regulated genes in cold-stress, but are not directly corresponding to any of the GO molecular functions and biological processes predicted from the differential miRNAs. Using several statistical models including a novel, network-based approach, we found that miRNAs identified in this study, either individually or together, and either directly or indirectly (i.e., mediated by transcription factors), only make minor contribution to the change in gene expression patterns under the low-temperature condition.

**Conclusions:**

Our results suggest that the cold-stress response of mRNA expression may be governed mainly through regulatory modes other than miRNA-mediated regulation. MiRNAs in animal brains might act more as developmental regulators than thermal adaptability regulators.

## Background

MicroRNAs (miRNAs) are a class of ~22 nt, non-coding RNAs present in plants and animals that play important roles in regulating gene expression by directing translational repression and/or mRNA destabilization [1-3]. Recognition and interaction between miRNA and mRNA take effect only if the 5' end seed (6-8 nt) of the miRNA is complementary to the 3' UTR site of the mRNA. A miRNA typically targets multiple, even hundreds of, genes [4], which makes miRNAs potentially global regulators responsible for spatio-temporal gene expression patterns.

Like *let-7 *[5,6], some miRNAs are highly conserved throughout evolution and participate in pivotal biological processes [7]. Lineage- or species-specific miRNAs may contribute to the uniqueness of organisms [8,9]. With recent advancements in miRNA microarrays and next-generation sequencing, miRNA expression profiles of many species are being generated at a rapid pace. These profiles are invaluable resources for better understanding gene expression dynamics [10-16]. Some miRNAs have been well characterized and have been shown to have important functions in normal development [17-21]. The deficient expression of some miRNAs also presents severe phenotypic abnormalities [22,23]. The role of miRNAs in responses to cellular and environmental stress has been discovered in a variety of organisms [24-28]. A few of these miRNAs have been characterized as key regulators of the stress response [25]. For example, miR-8 family miRNAs were shown to be indispensable in the response of zebrafish embryos to osmotic stress [29]. Despite this progress, most studies to date have only examined miRNAs' functions at individual level. The global contributions of microRNAomes (miRNAomes) to environmental adaptations remain largely unexplored.

The zebrafish, an important model organism, is an eurythermal fish with temperature tolerance ranging from approximately 7°C to 40°C [30], making it a useful experimental subject for exploring the molecular mechanisms underpinning thermal responses. Almost all of the previous work on the molecular underpinnings of thermal responses has focused on the fluctuated expression of protein-coding genes in response to warm or cold acclimation [31,32]. Many miRNAs have been discovered in zebrafish [33,34], and their expression profiles [35] and functions in developmental regulation have been studied [36]. Our goal was to determine how the zebrafish miRNAome responds to thermal acclimation and to assess the roles of miRNAs in thermally challenged transcriptomic plasticity. Toward this end, we systematically characterized the miRNAomes of zebrafish brains under normal and cold-acclimated conditions using Solexa sequencing. We also identified genome-wide gene expression patterns under each of these conditions using microarray profiling. We then examined the potential links between the changes in miRNA levels and the transcriptomic shifts and found that miRNAs may play minor roles in transcriptomic plasticity during cold acclimation in the zebrafish brain.

## Results

### Deep sequencing of the zebrafish brain small RNA and miRNA prediction

**Figure 1:**
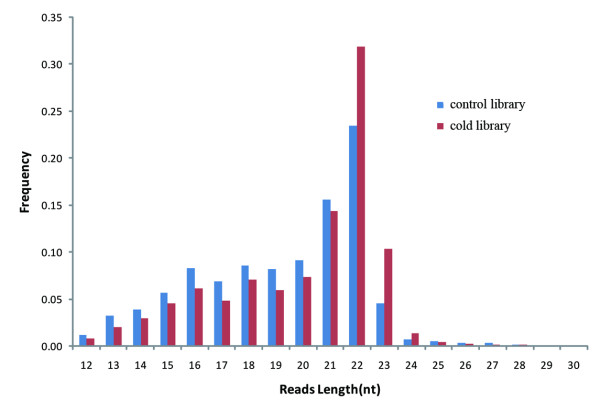
**Length distribution of the high-quality reads in the 10-30 nt range**.

Total RNAs from brain of cold-treated (10°C, 10 days) and normal control (28°C) zebrafish were extracted and electrophoresed to enrich the 18-30 nt small RNAs and then subjected to Solexa sequencing. The numbers of high-quality reads were 9,615,499 and 14,399,653 for the normal control (NC) and cold-acclimated (WC) RNA libraries, respectively. The length distributions of the sequence reads from the two samples both peaked at 22 nt, conforming to size distribution patterns characteristic of miRNA libraries (Figure 1). After filtering short (<18 nt) and adaptor-contaminated reads, we obtained 6,473,829 (NC) and 10,799,032 (WC) redundant reads that corresponded to 349,107 (NC) and 350,474 (WC) unique reads.

**Figure 2:**
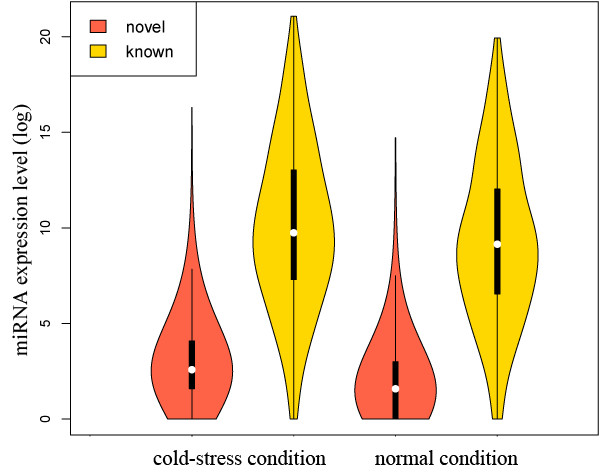
**Violin plot showing the expression levels of novel-identified and known miRNAs**. The violin plot combines box plot and kernel density trace to describe the distribution pattern of a vector of data. The four violin plots were drawn according to the expression levels of 525 newly identified and 175 known miRNAs under the normal and cold-acclimated condition, respectively. Note that if the expression level of a miRNA was 0 under certain condition, we assigned it to 1 in order to make the log-transfer of the raw data.

We ran the SOAP2 program [37] to map the unique reads to the zebrafish genome (Danio_rerio.Zv8.56.dna.toplevel.fa.gz). Stem-loop structures were predicted from the putative miRNA precursor sequences using MIREAP (https://sourceforge.net/projects/mireap/). Overall, we identified 602 stem-loop precursors that coded for 700 miRNAs. Among them, 175 were previously known miRNAs [38], constituting over 80% of the unique miRNAs (214) deposited in the miRBase database (release 16). 525 are newly identified miRNAs (Additional file 1). To cross-validate these newly identified miRNAs, we adopted another miRNA prediction software, miRDeep [39] by invoking the quantifier.pl script (using default parameters except that -g was set to 0) in the software package. Results showed that the miRNA prediction outcomes are almost the same, independent of the prediction methods used and the miRNA expression levels are also highly consistent between the two methods (Additional files 2, 3 and 4). To further evaluate the accuracy of miRNA prediction, we examined the predicted miRNAs with a higher stringency by adding another two criteria: 1) they cannot be exon-derived (a recent study by Stark *et al. *[40] suggested that miRNAs lie almost exclusively in the introns and intergenic regions of fruit flies); 2) their precursors need to be recognized as genuine stem-loop structures by the MiPred [41] software. Consequently, the number of newly identified miRNAs in our dataset was reduced from 525 to 399 (See Additional file 1). However, MiPred does not ensure 100% accuracy in determining the authenticity of miRNAs. For instance, 5 of the 175 well-known zebrafish miRNAs were incorrectly assigned in this prediction. In addition, "exon-derived" miRNAs are possible to exist if the relevant exons do not code for proteins or are transcribed under alternative promoters or from alternative strand. Therefore, the number of authentic miRNAs in the zebrafish brain is very likely higher than 574 (i.e., 175+399). To verify the authenticity of the newly identified miRNAs, we selected 13 miRNAs expressed at various levels to perform RT-PCR. All of them can be successfully amplified (Additional file 5). Cloning and sequencing of the PCR products confirmed that they are in correct forms. These results showed that the newly identified miRNAs are very likely true miRNAs. Remarkably, the newly identified miRNAs are expressed at substantially lower levels than previously known miRNAs (Figure 2). This may partially explain why the new miRNAs were missed by traditional cloning and sequencing methods.

Solexa sequencing are known for its short reads, and errors can be pervasive in short-read sequencing [42]. To address this concern, we clustered the raw reads using the FreClu software [43], which takes the read count into consideration. The results showed that this clustering process can improve the accuracy of short reads mapping (Table 1). However, it did not improve the data quality of the predicted miRNAs (Additional file 6). Hence, we performed all of the follow-up analyses of the miRNAs identified using the original reads.

**Table 1:** SOAP mapping results of reads with or without clustering.

		Before Clustering				After Clustering			
		**unique**	**%**	**redundant**	**%**	**unique**	**%**	**redundant**	**%**
**NC**	**# reads**	349107	-	6473829	-	255724	-	6473829	-
	**# matched**	102810	29.45	5016748	77.49	99235	38.81	5206070	80.42
**WC**	**# reads**	350474	-	10799032	-	287325	-	10799032	-
	**# matched**	153533	43.81	8680394	80.38	151896	52.86	8861106	82.05

WC: cold-acclimated condition; NC: normal condition.

### The miRNA expression profile in cold acclimation

**Figure 3:**
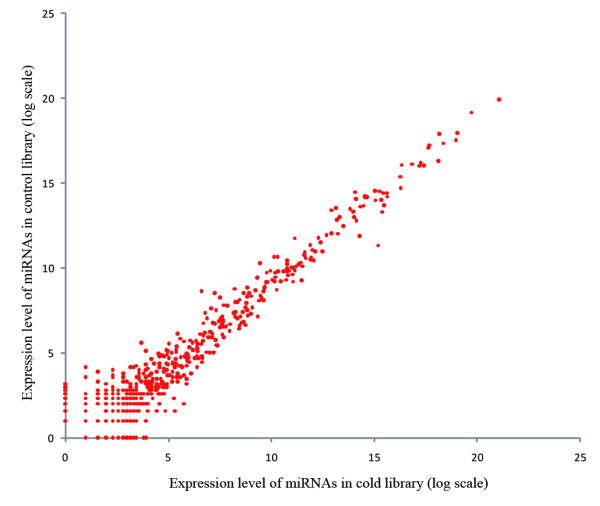
**Scatter plot showing the miRNA expression levels of the zebrafish cold and normal brain libraries**. The plot indicates much more expression noise of lowly expressed miRNAs than that of highly expressed ones. Hence, to define biologically significant miRNAs, we screened the differential genes from those with mean expression level greater than 64.

Based on normalized read counts of each miRNA in the control and cold libraries, we deduced the expression changes of the brain miRNAome in response to cold acclimation. Because low-expressed sequences have more noise than high-expressed sequences, Koh *et al. *proposed an adaptive thresholding method using Kolomogorov-Smirnov statistics to determine the threshold value for identifying significant miRNAs [44]. We observed a similar pattern in the present study. The less abundant miRNAs showed a greater divergence than the more abundant miRNAs (Figure 3). We hypothesized that the low-expressed miRNAs showing greater divergence likely have more noise than the high-expressed ones and that the high-expressed miRNAs have a high probability of playing an important biological role. We borrowed the concept of Koh *et al. *to define miRNAs differentially expressed under the normal and cold conditions as the ones that have a greater than 2-fold change between the two libraries and a mean expression level greater than 64. As a result, we found 11 up-regulated and 15 down-regulated miRNAs in the cold library; a majority of them are known miRNAs (Table 2).

**Table 2:** Differentially expressed miRNAs of zebrafish brain under cold-acclimated and normal conditions.

miRNA name	Genome coordinate	miRNA sequence	# WC	# NC	Fold△
**dre-mir-9-5-3p**	5:49144568:49144646:-	UAAAGCUAGAUAACCGAAAGU	37364	2559	7.377 (↑)
**dre-mir-30d-5p**	16:23860143:23860227:+	UGUAAACAUCCCCGACUGGAAGCU	19903	3770	2.667 (↑)
**miR_49**	4:52193679:52193757:+	UUCACUGUGGCGGAAAUGACC	146	31	2.380 (↑)
**miR_46**	21:25385786:25385876:+	AAGAGAAGAGUGAGCGAGUGA	149	32	2.352 (↑)
**dre-mir-30e-2-5p**	13:28550257:28550337:+	UGUAAACAUCCUUGACUGGAAGC	2856	617	2.339 (↑)
**dre-mir-24-4-3p**	22:5036700:5036779:+	UGGCUCAGUUCAGCAGGAACAG	652	142	2.320 (↑)
**dre-mir-2191-3p**	20:51108008:51108091:+	UCACACCUACAAUCCCUGGCA	435	99	2.221 (↑)
**dre-mir-146a-5p**	13:11738901:11738980:+	UGAGAACUGAAUUCCAUAGAUGG	42728	10063	2.145 (↑)
**miR_48**	24:36888097:36888173:+	UGAGGAGUUUAGAGCAAGUAA	129	31	2.102 (↑)
**miR_23**	5:8733083:8733158:-	AGCUGGUGUCCUGCAGAGUUU	345	85	2.051 (↑)
**dre-mir-455-5p**	5:66430757:66430834:-	UAUGUGCCCUUGGACUACAUC	168	42	2.021 (↑)
**dre-mir-30b-3p**	16:23860432:23860510:+	CUGGGCGGAGGGUGUUUGCU	456	462	2.006 (↓)
**dre-mir-145-5p**	14:40451735:40451816:+	GUCCAGUUUUCCCAGGAAUCCC	104	106	2.017 (↓)
**dre-mir-129-1-3p**	4:18861279:18861364:-	AAGCCCUUACCCCAAAAAGUAU	643	697	2.145 (↓)
**dre-mir-133a-2-3p**	23:5570884:5570961:-	UUGGUCCCCUUCAACCAGCU	117	130	2.199 (↓)
**miR_13**	5:6754333:6754409:+	UGUGGUGAUUAGAACAGGCUGA	159	181	2.253 (↓)
**miR_3**	Zv8_NA10078:51197:51280:-	AUGACUCAAACCCGAGGACUU	1279	1618	2.504 (↓)
**dre-mir-92a-1-3p**	8:45338553:45338629:+	UAUUGCACUUGUCCCGGCCU	9007	11736	2.579 (↓)
**dre-mir-458-3p**	14:26650559:26650634:-	AUAGCUCUUUGAAUGGUACU	17139	22528	2.601 (↓)
**dre-mir-129-2-3p**	7:50935695:50935777:-	AAGCCCUUACCCCAAAAAGCAU	7747	10824	2.766 (↓)
**dre-mir-152-3p**	3:21062159:21062235:+	UCAGUGCAUGACAGAACUUUG	2271	3467	3.021 (↓)
**dre-mir-183-5p**	4:14884980:14885059:+	UAUGGCACUGGUAGAAUUCACU	182	303	3.294 (↓)
**miR_5**	Zv8_NA3780:11797:11893:+	GCAUUGGUGGUUCAGUGGGA	698	1247	3.536 (↓)
**dre-mir-182-5p**	4:14886067:14886150:+	UUUGGCAAUGGUAGAACUCACA	151	365	4.783 (↓)
**miR_8**	2:35918353:35918422:+	AGCGGAUCAACUGAUGCGAC	98	394	7.956 (↓)

#WC: the number of miRNAs in the cold library; #NC: the number of miRNAs in the normal library; Fold△: normalized fold change of miRNAs in the normal over the cold library. ↑(↓) denotes the up(down)-regulation.

To reveal which biological processes might be regulated by the changed miRNAome, we predicted the target genes of the differential miRNAs using PITA [45]. We then projected the functions of these differential miRNAs onto the overrepresented Gene Ontology (GO) terms of their targets using the GO:TermFinder package [46] with a corrected p value of ≤0.05 as a cutoff. In total, we found 4 (dre-mir-30d-5p, dre-mir-182-5p, dre-mir-458-3p, and miR_8) and 5 miRNAs (miR_46, dre-mir-133a-2-3p, dre-mir-146a-5p, dre-mir-183-5p, and miR_8) with overrepresented biological process and molecular function GO terms, respectively (Table 3). In addition, we assembled all target genes of the up-regulated and down-regulated miRNAs and examined whether any overrepresented functions would emerge. Our results showed that genes related to "purine nucleotide binding" and "nucleotide binding" were enriched in the targets of up-regulated miRNAs.

**Table 3:** Differentially expressed miRNAs and their functions.

miRNA name	Fold△	Molecular Function	Biological process
**dre-mir-30d-5p**	2.667 (↑)	ND	Phosphoinositide metabolic process
**miR_46**	2.352 (↑)	ribonucleotide binding, ATP binding	ND
**dre-mir-146a-5p**	2.145 (↑)	protein tyrosine/serine/threonine phosphatase activity	ND
**dre-mir-182-5p**	4.783 (↓)	ND	glucose metabolic process
**dre-mir-458-3p**	2.601 (↓)	ND	regulation of developmental process
**miR_8**	7.956 (↓)	aminoacyl-tRNA ligase activity	tRNA aminoacylation for protein translation
**dre-mir-133a-2-3p**	2.199 (↓)	receptor signaling protein activity	ND
**dre-mir-183-5p**	3.294 (↓)	cytoskeletal protein binding	ND

ND: Not determined. Fold△: fold change of miRNAs in the cold over the normal library. ↑(↓) denotes the up(down)-regulation.

### Gene expression profiling and differentially expressed genes

Gene transcription changes between the cold-acclimated and normal samples were obtained by microarray-based hybridization using three sets of RNA samples prepared from fishes of three acclimation experiments. The gene expression level was measured as the intensity of probe sets on an Affymetrix microarray. After filtering the probe sets and retrieving the map relationship between the probe sets and the genes, we obtained gene expression profile recordings of 7356 protein-coding genes. The differential genes were determined using a two-class, unpaired comparison in the SAM (version 3.0) program [47]. Using a 5% false discovery rate (FDR) cut-off, we found that 311 genes were down-regulated and 315 genes were up-regulated in the cold-acclimated samples (Additional file 7). There were 127 and 85 genes with more than 2-fold changes in mRNA abundance that were down- and up-regulated, respectively.

We then investigated the functional enrichment of these differential genes using the GO:TermFinder package [46]. As shown in Table 4, under cold stress, the genes with a functional annotation related to "mRNA processing" were enriched in the up-regulated differential genes, and genes associated with "cellular macromolecular complex subunit organization" were overrepresented in the down-regulated genes. An interesting observation here is that the identified molecular functions and biological processes changes in the cold-acclimated zebrafish brain bear no apparent overlap with those predicted from the potential targets of the differential miRNAs (see Table 3), suggesting the role of miRNAs might be minor in the transcriptomic changes in cold stress. We thus examined the possible regulation roles of the microRNAome in cold adaptation by other statistical approaches.

**Table 4:** Functional enrichment of differentially expressed mRNAs.

Category	# genes	Molecular Function	Biological process
**Up-regulated**	315	nucleotide binding	mRNA processing
**Down-regulated**	311	ND	cellular macromolecular complex subunit organization
**Up-regulated & >2-fold change**	85	ND	response to stress response to temperature stimulus
**Down-regulated & >2-fold change**	127	dipeptidase activity oxidoreductase activity	cellular macromolecular complex assembly nucleosome assembly

# genes: the number of genes in the category; ND: not determined.

### Verification of the expression pattern of the protein-coding genes and miRNAs by quantitative RT-PCR (q RT-PCR)

**Figure 4:**
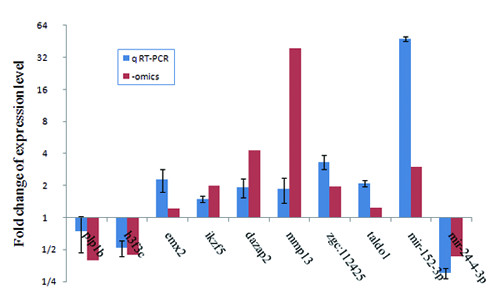
**Fold change of expression level for selected genes and miRNAs measured by q RT-PCR**. Quantitative RT-PCR shows that the direction of expressional change under the cold condition of the selected genes and miRNAs correlated nicely with the results obtained from the "-omics" data (Additional file 7 and Table 2).

The reliability of statistical analyses depends on the quality of the microarray and solexa profiling data. To evaluate the accuracy of the microarray screening and the miRNA profiling results, genes and miRNAs with change direction either down-regulated (*plp1b*, *h3f3c *and dre-mir-24-4-3p) or up-regulated (*emx2*, *ikzf5*, *dazap*, *mmp13*, *zgc:112425*, *taldo1*, and dre-mir-152-3p) were selected for quantitative RT-PCR verification. Q-PCR showed that the direction of expressional change for each selected gene correlated perfectly with the results of the microarray screening or solexa sequencing (Figure 4). Although the changed folds of expression in a few genes (*mmp13a*, *plp1b*) and the dre-mir-152-3p showed some discrepancy between the q-PCR results and the '-omic' (microarray and solexa sequencing) profiling data, the other genes/miRNAs are in good agreement. Since we evaluate only the direction of change of the genes and miRNAs when delineating the potential relationship between miRNA-mediated regulation and transcriptomic shifts (see later sections), the gene and miRNA expression profiles obtained from the '-omic' profiling approaches are generally useful for statistical analyses.

### Statistical tests for the potential correlation between the expression levels of miRNAs and protein-coding genes

Mounting evidence has shown that miRNAs can orchestrate the expression of mRNAs and that the well-balanced interplay between the miRNAome and protein-coding gene expression plays a crucial role in an organism's development [48-50]. To investigate the influence of miRNAs on the global expression of protein-coding genes in cold stress, we adopted four different approaches. First, we estimated the effect of miRNAs on differentially regulated genes using Fisher's exact test. Using PITA target prediction methods, we obtained 18665 pairs of miRNA-target interactions in total. Our results showed only one miRNA (dre-mir-34-3p) with target genes overrepresented in the down-regulated gene set. No miRNAs showed targets enrichment or depletion in the up-regulated gene set (FDR < 5%). The miRNA dre-mir-34-3p was expressed at very low levels (29 in the cold library and 20 in the control library) in the brain and was not significantly different between the two conditions. We speculate that it is not probable that dre-mir-34-3p plays an important role in the changed gene expression patterns observed under cold stress. To address concerns over potential target prediction bias by PITA, we performed target prediction by miRanda software [51]. Under this methodology, we obtained 56670 pairs of miRNA-target interactions, which is about 3-fold of the number (18665) obtained by PITA prediction method. We then examined the composition of targets predicted by the two methods for each miRNA. Our results showed that 615 miRNAs shared at least one predicted target between PITA and miRanda; however, as a whole, the overlap is relatively low (Additional file 8, Table S3-S6). For example, among a group of 370 miRNAs for which each has more than ten targets by whatever target prediction methods, the median value of the proportion of overlap is only 0.396 (Additional file 9, Figure S3). In average, each miRNA targets ~80 protein-coding genes and each gene is likely to be regulated by 7-8 miRNAs in return. Based on these interaction data, our statistical analyses recaptured the fore-mentioned miRNA-mRNA interacting pattern, i.e., no miRNAs targeted over- or under-represented genes in either up- or down-regulated gene set.

Recently, Chen *et al. *[52] proposed a novel concept for the regulatory effect score (RE score) to measure the inhibition capability of a miRNA on its targets. In principle, the RE score is calculated as the average difference in expression levels between a target and a non-target group. By calculating the RE scores on the PITA and miRanda methodology, respectively, we checked whether some of the miRNAs would changed regulatory effects on their targets between the two conditions. Consistent with previous results, we again found that no miRNAs exhibited a significant change in their regulatory effect after correcting the raw t-test p-values by the multiple hypothesis test (FDR < 0.05) whatever the target prediction method was used. Taken together, our results indicate that individual miRNAs are unlikely to be major factors leading to the changed transcriptomic phenotype of zebrafish brain under cold stress.

To further reduce the concerns over target-prediction bias induced errors, we applied the Sylamer algorithm [53] to re-examine the potential relationship between the miRNAome and transcriptome under cold acclimation. We sorted the protein-coding genes from the most significantly up-regulated to the most down-regulated. We then calculated the distribution of the enrichment/depletion of words in the 3' UTR sequences that could be complementary to the seed sequences of miRNAs. Since positions 1-7 and 2-8 of the 5' end of a mature miRNA are the most important sequences for target recognition [54], we took the 7 nt region as a miRNA seed in this study and examined whether some over- or under-represented words corresponded to certain miRNAs.

**Figure 5:**
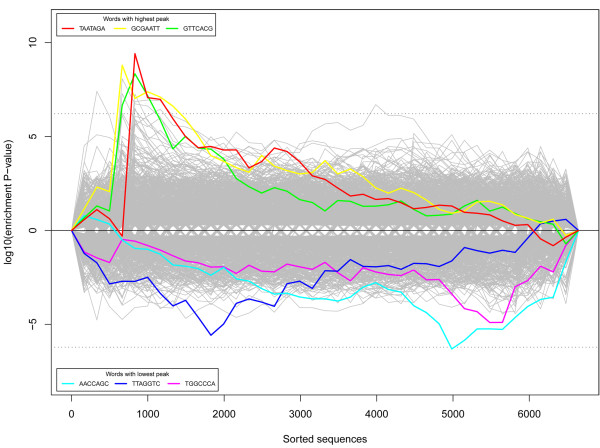
**The word enrichment and depletion pattern in mRNA 3' UTR**. Genes are sorted by their expression divergence between normal and cold-acclimated zebrafish (from the most up-regulated to the most down-regulated). Probability values with a plus-sign are enriched; those with a minus-sign are depleted.

As shown in Figure 5, there are no obvious peaks in the region containing the most down-regulated genes, suggesting that the analysis failed to identify any miRNAs being the major and direct factors contribute to the decreased expression levels of the down-regulated gene sets; on the other hand, few than ten words seem to be overrepresented in the 3' UTR regions of the up-regulated genes. The three most overrepresented words are TAATAGA (P = 3.86 × 10^-10^), GCGAATT (P = 1.58 × 10^-9^) and GTTCACG (P = 4.58 × 10^-9^). However, these words are not complementary to any seed region of the profiled zebrafish miRNAs. We then examined the composition of the top 10 most enriched words and found only one, GCTAGTT (P = 5.53 × 10^-8^), that was complementary to the seed region of the newly identified miR_381 (**AACTAGC**AGCTGTTGACATCCA). However, this miRNA was hardly expressed in the brain (1 in the control library and 4 in the cold library), suggesting that it was unlikely to be the major factor responsible for the changed gene expression patterns observed.

**Figure 6:**
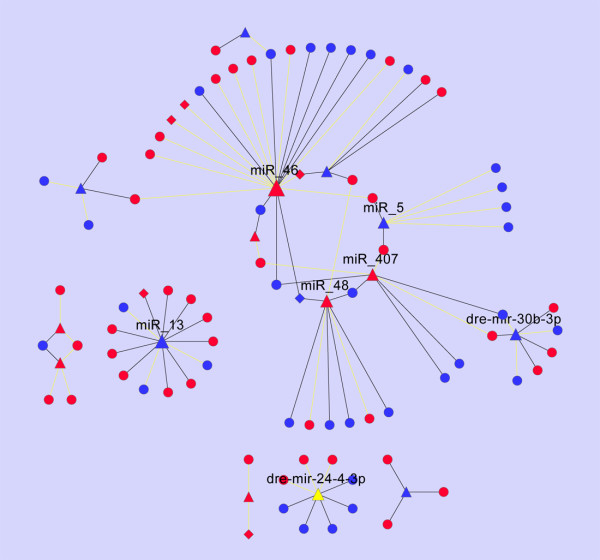
**Graphic illustration of the regulatory relationship between the differential miRNAs and mRNAs predicted by PITA**. Triangles, circles and diamonds denote miRNAs, non-TF genes and TF genes, respectively. The nodes in red and blue denote up- and down-regulated miRNAs or mRNAs, respectively. The edges in dark and yellow denote coherent and incoherent regulation, respectively. The size of a node is proportional to the number of its partners. Of nodes with degree > 5, the associated name is shown.

To systematically investigate the extent to which miRNAome-mediated regulation could have influenced the transcriptome under cold-acclimation, we designed a novel, network-based methodology to parse the subtle regulatory effects of a group of miRNAs on a group of protein-coding genes at the system level. Based on the predicted miRNA-target relationship predicted from the PITA method, we first built a miRNA-mRNA network of 32 differential miRNAs and 626 mRNAs (311 down-regulated and 325 up-regulated). The miRNA-target relationship showed that the majority of the differential gene transcripts do not possess any potential targeting sites in the 3' UTRs that correspond to the differentiated miRNAs. In addition, approximately half of the differential miRNAs do not target any genes in the differentiated gene list. The resultant miRNA-mRNA network involved only 97 nodes (82 mRNAs and 15 miRNAs) and 97 edges when the isolated nodes were separated. As seen in Figure 6, the miRNA-mRNA interaction networks are composed of six unconnected components. One component contains 60 of the 97 nodes, whereas the others are formed by only 1 or 2 miRNAs situated in the network center. The node with the highest number of edges is the up-regulated miRNA miR_46 that was predicted to target 18 (11 up-regulated and 7 down-regulated) gene transcripts. Among the 82 miRNA-targeted differentially expressed genes, over four-fifths (67/82) are predicted to be targeted by only 1 miRNA and one-fifth by 2 or more. We then computed the "regulatory density" (RD) of the network by formula (1) (see Methods). We developed this formula and define it as a proxy for the regulatory potential of a group of miRNAs upon gene transcripts by calculating the proportion of coherent over incoherent regulatory links within a miRNA-mRNA network. Our analysis showed that the observed RD was 0.1134. However, the values of the 95th- and 99th-quantiles were 0.1546 and 0.1959, respectively, for the 1000 cohorts of random networks that were constructed by shuffling the original miRNA-mRNA network while maintaining the degree distribution. We further analyzed the topologic property of a similar network derived by using the miRanda prediction data. The produced miRNA-mRNA network consisted of 203 edges that link 25 and 161 differential miRNAs and protein-coding genes, respectively. As seen in Additional file 10, a majority of the mRNAs are targeted by a few miRNAs; and only a few miRNAs have >5 target genes. Calculation showed that the RD score of this network is 0.0148, again not significantly greater than random expectation (empirical P-value: 0.269). Taken together, results from all the above statistical analyses suggested that the miRNAome is unlikely to be the major regulatory component responsible for the changed expression profiles of the protein-coding genes under cold stress.

Mathematical models [55] and statistical associations [56,57] have shown that miRNAs frequently intertwine with transcription factors (TFs) in regulatory circuits and play essential roles in signal transduction. Recently, Tu *et al*. [58] proposed a two-layer network wherein miRNA-initiated regulation was mediated and propagated through the miRNAs' targeted transcription factors. We investigated whether the miRNAs had indirectly, through their transcription factors, led to the observed transcriptomic plasticity. We first filtered the transcription factors from the arrayed gene list according to the GO annotation. We treated the genes with any one of the GO terms, "DNA binding", "transcription factor activity", "transcription activator activity" or "transcription repressor activity", as putative transcription factors. Accordingly, we predicted 796 TF-coding genes among the 7356 protein-coding genes. Notably, only 46 of these TFs showed differential expression in cold acclimation, and they were significantly underrepresented in the differential expressed gene list (46 out of 626 differential genes versus 796 TFs in the 7356 arrayed genes, Fisher's exact test: P < 0.01). Among the differential TF genes, only 6 were predicted to be the targets of 5 differential miRNAs (the ◆-▲ pair in Figure 6). Of the 6 TFs, 3 showed coherent regulatory direction with their respective miRNA partners (miR_13→*emx2*; dre-mir-183-5p→*ikzf5*; miR_46, miR_48→*h3f3c. *see Figure 6). Given that the gene *emx2 *represented slightly up-regulatoin (fold-change: 1.214; q-value: 4.376%) under cold-acclimated condition, we speculate that it was not a driven gene responsible for the changed mRNA expression pattern. There is no evidence that the *ikzf5 *(fold-change: 1.994; q-value: 0) and *h3f3c *(fold-change: 2.245; q-value: 2.144%) are related to stress response when we examined their annotations in multiple databases and in the literature. Additionally, almost no differential TF-coding genes were targeted by differential miRNAs. Taken together, we conclude that the mRNA phenotypic plasticity seen under the cold environment was not dominantly modulated by miRNAs, even if these factors were directly or indirectly mediated by transcriptional factors.

## Discussion

The repertoires of miRNAs have been constantly revised and updated with the advance of *in silico *prediction methods and more intensive cloning and sequencing efforts in broader tissues and developmental stages. The number of unique, mature human miRNAs is now up to 1223 in the miRBase database (version 16). In contrast, there are only 214 mature miRNAs now registered for zebrafish. This relatively low number could be the result of genomic between-species differences or incomplete miRNA identification in zebrafish. By Solexa sequencing, we comprehensively profiled the miRNA repertoire in the zebrafish brain. The number of predicted miRNAs in this tissue reached 700 (574 in high-stringency criteria) in this study and greatly expanded the miRNAome in zebrafish. Of note is that the expression levels of the newly identified miRNAs in the zebrafish brain are mostly low. The authenticity of these newly identified miRNAs remains to be further investigated to determine their biological functions. However, a population analysis on 7 randomly selected novel miRNAs currently carried out in the laboratory showed that almost half of them are subjected to purified selection suggesting that many newly identified miRNAs may have high potentials to be functional *in vivo *despite their low expression levels. It is possible that some of these newly identified, low-expressed miRNAs could be expressed at higher levels in tissues other than the brain and play roles in gene regulation. The miRNAome of the zebrafish is much larger than those of the lower chordates such as the amphioxus species, which had been determined by small RNA library sequencing [59] and solexa sequencing [13]. This suggests the expansion of the miRNA regulome in the evolution of vertebrates.

**Figure 7:**
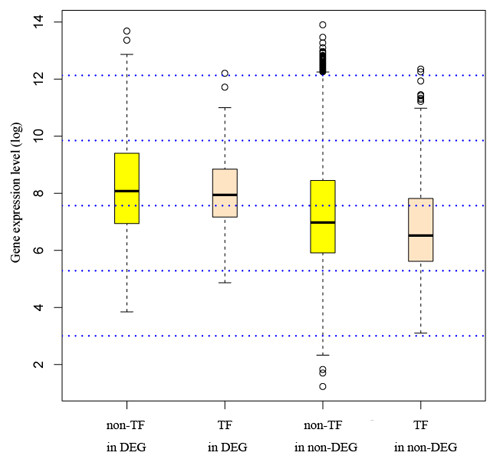
**The expression pattern of TFs and non-TFs within differential or non-differential gene sets**. The boxplot shows that the TFs expressed at a slightly lower level than the non-TFs. However, the expression pattern for TFs as compared to non-TFs within the differential genes is very similar as that within the whole set of the arrayed genes, indicating that the relatively low expression of TFs did not influence the screening of the differential TF genes in this study. DEG: differential genes; non-DEG: non-differential genes.

To study the function of the miRNAome in environmental adaptation, we designed the cold-acclimation experiments in this study. We profiled both the miRNAome and the transcriptome of the brain under low- and normal-temperature conditions. We adopted four approaches to examine how miRNAs, either individually or globally, could have influenced the transcriptomic plasticity under cold conditions. Using statistical methods we first examined the potential effects of individual miRNAs on gene expression patterns. We found one or two miRNAs exhibiting regulation signatures on mRNA expression based on enrichment/depletion analysis of target genes among the differential gene set. However, we argue that these miRNAs are unlikely to have been responsible for the different expression patterns after further investigation of their expression levels. Our results show that at the individual level, the miRNAs were not a main factor shaping the mRNA expression patterns observed under the cold-acclimated conditions. We then attempted to clarify whether the cold-stress response of mRNA expression is governed by miRNAs indirectly and is mediated by TFs. TF-mediated transcriptional regulation is thought to be stronger than the post-transcriptional regulation usually mediated by miRNAs [60]. For this purpose, we assigned a set of TFs according the GO annotation and found that TFs were significantly underrepresented in the differential protein-coding genes; there were only 6 TFs targeted by differential miRNAs. Again, our results suggest that there is hardly any influence from miRNAs on the changed mRNA expression patterns. A recent study by Nunez-lglesias *et al. *[50] suggested that a subtle regulation relationship between miRNAs and their targets can be observed in the global miRNAs-mRNA network using more sensitive experimental designs and a systems biology approach. Aiming to discern the subtle regulatory effects, we employed a novel network-based analysis. We found that the miRNA-mRNA network (formed by the regulatory relationship between differential miRNAs and differential mRNAs) does not possess a significantly higher percentage of coherent edges than the random expectation. Furthermore, this regulatory network consists of only 3 pairs of coherent edges between miRNAs and TF genes. Therefore, our data also rule out the possibility that miRNAs as major factors indirectly modulate mRNA expression in the brain of cold-acclimated zebrafish. One might argue that many TFs are often expressed in low abundance and that some of them might go beyond the detective sensitivity of the current Affymetrix array screening methodology, causing the observed underrepresentation of the TFs in the differential gene set. This situation might eventually lead to incomplete evaluation of the regulatory effects of miRNAs on the transcriptome. To evaluate this possibility, we calculated the mean expression level of the 796 predicted TFs and the non-TF genes in the array. As seen from figure 7, though the TFs showed slightly lower average expression levels than the non-TF genes, the distribution pattern of expression intensity for TFs as compared with non-TFs within the differential genes is very similar as that within the whole set of the arrayed genes, indicating that the relatively low expression of TFs did not hinder the screening of the differential TF genes in this study. Evidence from various developmental biology studies has been continuing emerged favoring the hypothesis that miRNAs are capable of imparting robustness on gene regulation networks that involve several classes of recurrent circuits composed of miRNAs and their corresponding targets [61]. For example, a study by Hilgers *et al. *[62] revealed that miR-263a/b plays an important role in protecting *Drosophila *mechanosensory bristles from apoptosis and ensures the developmental robustness of this organ. In our study, we found miRNAs are minor factors in transcriptome changes in the cold acclimated zebrafish brain. This difference may reflect that miRNAs in animal brains act more as developmental regulators than adaptability regulators.

Thermally invoked responses can be generally classified as cellular stress responses, homeostatic responses or body remodeling-related responses that correspond to short-term, mid-term or long-term acclimation processes, respectively [63-65]. Our samples were taken after 10-day cold acclimation, which can be classified as mid-term acclimation. We reason that the current results reflect only the gene regulatory patterns of the zebrafish brain at this temporal scale of acclimation. The involvement of miRNAs as a pivotal regulator in an earlier or later cold adaptation phase remains to be further investigated.

Persistent efforts have identified and characterized some cold stress-induced protein-coding genes [66,67]; among them, *rbm3*, which encodes a glycine-rich RNA binding protein, has been suggested to regulate global protein synthesis by affecting miRNA expression levels [68]. In the present study, we also observed two homologous genes in zebrafish, *cirbp *(fold-change: 2.244; q-value: 0) and *zgc:112425 *(fold-change: 1.961; q-value: 1.598%), with significantly up-regulated expression levels under cold-acclimated condition.

Lopez-Maury *et al. *[69] have pointed out that post-transcriptional regulatory mechanisms, compared to transcriptional mechanisms, are largely unknown under stress conditions. To our knowledge, there are few studies jointly analyzing miRNAs and mRNA profiles to explore the roles of miRNAs on the cold adaptation of fish. Such a study would extend our understanding of miRNAs and environmental adaptation and undoubtedly lead to a more challenging and interesting question: whether and how miRNAs have integrated into cold-adaptive processes and contributed to the unique physiology of fishes endemic to extreme temperature conditions.

## Conclusions

In this study, we systematically characterized the miRNAome of zebrafish brain under normal and cold-acclimated conditions using Solexa sequencing. Our data has greatly extended the zebrafish miRNAs repertory from the 214 unique, mature miRNAs (miRBase databse, release 16 version) to more than 574 ones. Besides, our results showed that the miRNAs, either individually or together, either directly or indirectly play only a minor role in transcriptomic plasticity during cold acclimation in the zebrafish brain, suggesting that the cold-stress response of mRNA expression may be governed mainly through regulatory modes other than post-transcriptional regulation by miRNAs. MiRNAs in animal brains may act more as developmental regulators than thermal adaptability regulators.

## Methods

### Zebrafish treatment and brain RNA preparation

Adult zebrafish (*Danio rerio*) were alternatively divided into two groups: a control group and an experimental group. In each group, the sex ratio was 1:1. The control group was raised under the normal laboratory breeding temperature (28°C), and the experimental group was subjected to cold (10°C) stress. An abrupt transfer of zebrafish from normal to cold (10°C) environments will cause most of them to die. To prevent this, the temperature of the cold group was decreased from 28°C to 10°C at a rate of one degree per hour. After 10 days of cold adaption, the brains of the zebrafish were dissected, and total RNAs were extracted from these brains using the total RNA isolation kit (Promega, Madison, USA). All experimental research on animals was conducted with the approval of the Animal Research Ethics Committee of our institute.

### Microarray profile

To evaluate the genome-wide change of gene transcription in cold stress, we performed hybridization analysis using the Zebrafish Genome Array (Affymetrix) as previously described [70]. Briefly, mRNAs were isolated from 10 μg of total RNAs derived from pooled zebrafish brains of acclimated and control samples using the Eukaryotic Poly-A RNA Control Kit (Affymetrix) in an RNase-free environment. Subsequently, mRNAs were subjected to double-strand cDNA synthesis using a one-cycle cDNA synthesis kit (Affymetrix). The resulting double-strand cDNAs were *in vitro *transcribed into biotin-labeling cRNAs using the IVT Labeling kit (Affymetrix). After that, biotin-labeling cRNAs were fragmented by heating and hybridized with the Affymetrix Zebrafish Genome Arrays. Finally, the signal intensity of the chip was scanned using the Gene Chip Scanner 3000 (Affymetrix) and analyzed with the Gene Chip Operating Software (Affymetrix). The data derived from the genome-wide expressional analysis was deposited in the GEO bank with the accession number **GSE32092**.

### Small-RNA deep sequencing

For small-RNA Solexa sequencing, 20 μg of total RNAs were subjected to electrophoresis on a polyacrylamide gel under denaturing conditions. The small RNAs ranging from 18-30 nt were excised and purified. The resulting small RNAs were sequentially ligated to a 5' adaptor and a 3' adaptor. The products were then reverse transcribed (RT) and amplified by polymerase chain reaction (PCR). The RT-PCR products of approximately 90 bp (small RNA + adaptors) were isolated and subjected to sequencing analysis using the Illumina Genome Analyzer (Illumina, San Diego, USA) according to the manufacturer's instructions.

### MiRNA identification and annotation

High-quality, small-RNA reads larger than 18 nt were extracted from raw reads and mapped to zebrafish genome sequences (Danio_rerio.Zv8.56.dna.toplevel.fa.gz) using SOAP2 [37]. The miRNAs and their precursor structures were predicted by MIREAP (https://sourceforge.net/projects/mireap/) under the default settings using the perfect-matched reads. Like the miRDeep software [39], MIREAP integrates miRNA biogenesis, sequencing depth and structural features to identify miRNAs and further interrogate their expression level from deep sequenced small RNA libraries. Stem-loop hairpins were retained only when they comply with: 1) the mature miRNAs-associated reads are mapped in the arm region of the precursors; 2) the free energy of the secondary structure calculated by RNAfold [71] is lower than -18 kcal/mol. In detail, the potential mature miRNA is defined as the most abundant read sequence that aligns to the potential precursor sequence; the expression level of a miRNA is then specified as the sum of an ensemble of reads that align with the potential mature molecules, allowing three nucleotides sliding beyond the position of the potential mature miRNA at the 5' end. We then performed blast analyses on the predicted miRNAs precursors and mature miRNAs against sequences deposited in the miRBase (release 16) database to annotate these miRNAs. Novel miRNAs were further tagged as "exon-derived", "intron-derived" or "intergenic" miRNAs according to the loci of their precursors on the genome. In addition, MiPred [41] was used to distinguish between genuine and pseudo miRNA precursors. This software incorporates the local, contiguous structure-sequence composition and the minimum free energy (MFE) of the secondary structure to eliminate potential false positive predictions.

### Frequency-based small-RNA reads clustering

Following the method proposed by Qu *et al*. [43], the raw reads were clustered according to their read numbers. After clustering, the accuracy of mapping short reads can be improved.

### Gene expression validation by q RT-PCR analysis

Total RNA (8 μg) from the cold and normal fishes was respectively reverse transcribed by Takara PrimeScript RT-PCR Kit (Takara, CN) with OligodT primers, according to the protocol the manufacturer recommended. Another 4 μg total RNA from the two treatments was used for small RNA (< 200 bp) isolation by Ambion mirVana miRNA Isolation Kit. Poly (A) tail was added to the small RNAs by Ambion Poly (A) Polymerase (Ambion, CN). 2 pairs of primers for each selected gene or miRNA were designed and tested for amplification efficacy and specificity by agarose electrophoretic analysis of the PCR products. The primer pair with the best specificity was selected for further use in q-PCR. Q-PCR was run by using the SYBR RT-PCR kit in a Bio-rad CFX 96 (Bio-rad) machine and results were analyzed by the CFX Manager software. The PCR thermal cycling was performed using 45 cycles of 95°C for 15 seconds, 72°C for 30 seconds and 60°C for 30 seconds. The PCR reaction for each gene was performed in triplet with either the housekeeping gene Beta-actin (for arrayed genes) or U6 RNA (for miRNAs) as control.

### Word enrichment/depletion in 3' UTR

The mRNAs were sorted according to their significance of expression levels and their corresponding 3' UTRs. The longest sequences were retrieved from the Ensembl website using Biomart tools [49]. The word enrichment/depletion patterns of these genes were generated by Sylamer [53].

### Regulatory effect score

The regulatory effect score (RE score) was defined as the inhibitory effect of a miRNA on the mRNAs in a sample, according to the average difference in expression of its target versus non-target mRNAs [52]. For each miRNA, we first calculated the rank-based RE score for every sample and then compared the difference between the normal and cold-acclimated groups using a two-sample t-test. The FDR values were calculated using the R multtest [72] package.

### GO enrichment test

For the list of mRNAs, we tested whether each had enriched GO terms in biological processes and molecular functions using the GO:TermFinder package [46]. The GO annotation file was downloaded from http://zfin.org/zf_info/downloads.html#go/on January 29, 2010. The GO ontology file was downloaded from http://www.geneontology.org/ on January 23, 2008.

### miRNA target predictions

Currently, there are several popular tools for predicting miRNA targets, including PITA [45], PicTar [73], miRanda [51], mirWIP [74] and TargetScan [75]. These tools can be generally divided into two classes, methods that integrate the information of sequence conservation across multiple genomes and those that do not take this into account. Because of the limited genome data for zebrafish relatives, we predicted the miRNA targets by two methods, PITA and miRanda, independently. PITA scores the miRNA-target interactions based on a thermodynamic model. A salient feature of this program is that it considers the site accessibility of the surrounding mRNA sequence but disregards sequence conservation between genomes. Thus, non-conserved or even species-specific target genes could be identified using this method. For each miRNA, the target genes are defined as those predicted to contain at least one binding site (△△G ≤ -10) at their 3' UTR. We also ran miRanda (August 2010 Release) to predict the target genes with default parameters except that the energy parameter is set to -20.

### Network analysis

The miRNA-mRNA relationship is modeled as a bipartite where an edge links a miRNA and an mRNA with a predicted binding site in its 3' UTR corresponding to the miRNA. We defined coherent and incoherent edges as those for which their associated miRNAs and mRNAs represented the opposite and parallel expression change under cold stress, respectively. We then calculated a regulatory density (RD) for a group of miRNAs and mRNAs within a network according to the following formula:

(1) RD=(#CE-#IE)∕(#CE+#IE),

where #CE (#IE) denotes the number of coherent (incoherent) edges.

To estimate the significance of the observed RE, we generated 1,000 cohorts of random networks and recalculated the RE by shuffling the original network while keeping the degree preservation according to the published method [76].

## Authors' contributions

RY, ZD and LC conceived of and designed the study; ZD and SC performed the experiments; RY analyzed the data; RY and LC wrote the paper. All authors read and approved the final manuscript.

## Supplementary Material

Additional file 1 **Table S1. List of 525 newly identified zebrafish miRNAs**. Click here for file

Additional file 2 **Figure S1. Scatter plot shows a high degree of consistency between miReap and miRDeep on miRNA identification**. Click here for file

Additional file 3 **Data S1. Newly identified miRNAs and their precursors in normal zebrafish brain**. The predicted secondary structures (bracketed notations) were produced by miRDeep2. The reads that were mapped to the precursors are aligned below, with the number of reads shown on the left. Click here for file

Additional file 4 **Data S2. Newly identified miRNAs and their precursors in cold-acclimated zebrafish brain**. The method used here is the same as stated in Additional file 3. Click here for file

Additional file 5 **RT-PCR verification of newly identified miRNAs**. Click here for file

Additional file 6 **Figure S2. The same pattern holds for miRNA species and expression levels with or without frequency-based reads clustering**. Click here for file

Additional file 7 **Table S2. List of significantly up- or down-regulated genes**. Genes with 2-fold changes are highlighted. Genes in red and blue are up- and down-regulated genes under the cold-acclimated condition. Click here for file

Additional file 8 **Table S3. List of miRNA-target pairs predicted by PITA. Table S4. List of miRNA-target pairs predicted by miRanda. Table S5. List of miRNA-target pairs predicted by PITA and miRanda. Table S6. Summary of target overlap between PITA and miRanda**. Click here for file

Additional file 9 **Figure S3. Histogram showing the distribution of miRNA targets commonly predicted by PITA and miRanda**. 370 miRNAs were counted, each of which targeted more than 10 genes by whatever prediction methods used. The degree of overlap is calculated as the ratio of the number of shared targets to the minimal value of the number of targets predicted by PITA and miRanda, separately. Click here for file

Additional file 10 **Figure S4. Graphic illustration of the regulatory relationship between the differential miRNAs and mRNAs as predicted by miRanda**. The style of this graph is the same as Figure 6. Click here for file
