# Supplementary figures and images for: MicroRNA-mediated gene regulation plays a minor role in the transcriptomic plasticity of cold-acclimated Zebrafish brain tissue
Source: BMC Genomics. 2011 Dec 14;12:605. doi: 10.1186/1471-2164-12-605 (PMC3258298; doi:10.1186/1471-2164-12-605)

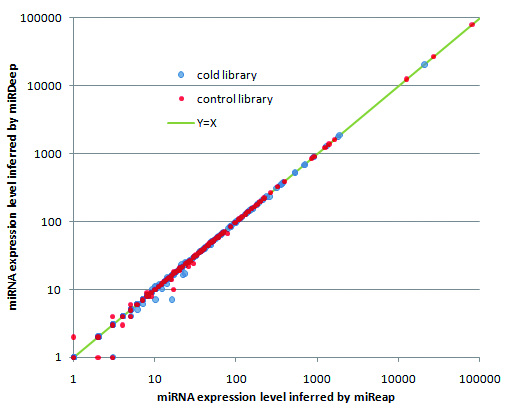

Supplement: Additional file 2 — Figure S1. Scatter plot shows a high degree of consistency between miReap and miRDeep on miRNA identification. [file 1471-2164-12-605-S2.TIFF]

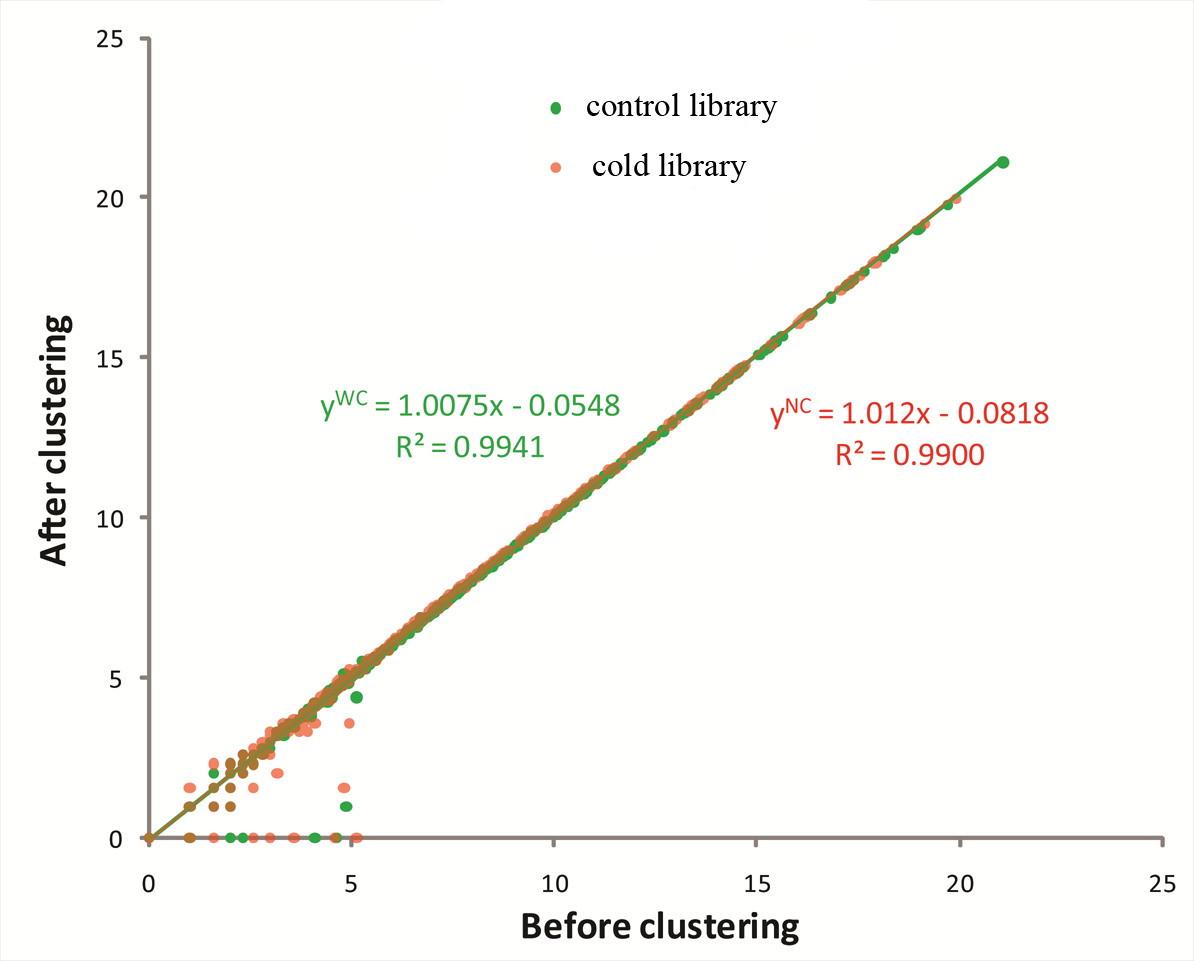

Supplement: Additional file 6 — Figure S2. The same pattern holds for miRNA species and expression levels with or without frequency-based reads clustering. [file 1471-2164-12-605-S6.TIFF]

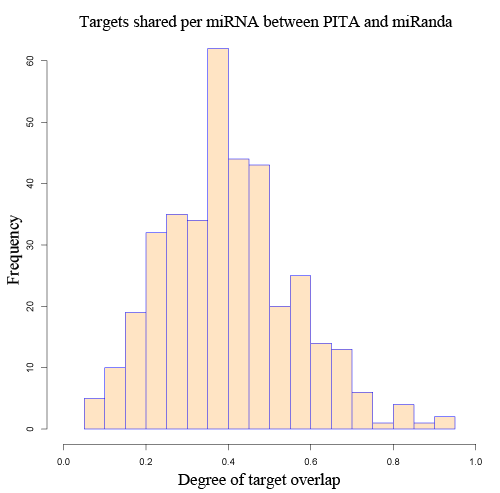

Supplement: Additional file 9 — Figure S3. Histogram showing the distribution of miRNA targets commonly predicted by PITA and miRanda. 370 miRNAs were counted, each of which targeted more than 10 genes by whatever prediction methods used. The degree of overlap is calculated as the ratio of the number of shared targets to the minimal value of the number of targets predicted by PITA and miRanda, separately. [file 1471-2164-12-605-S9.TIFF]

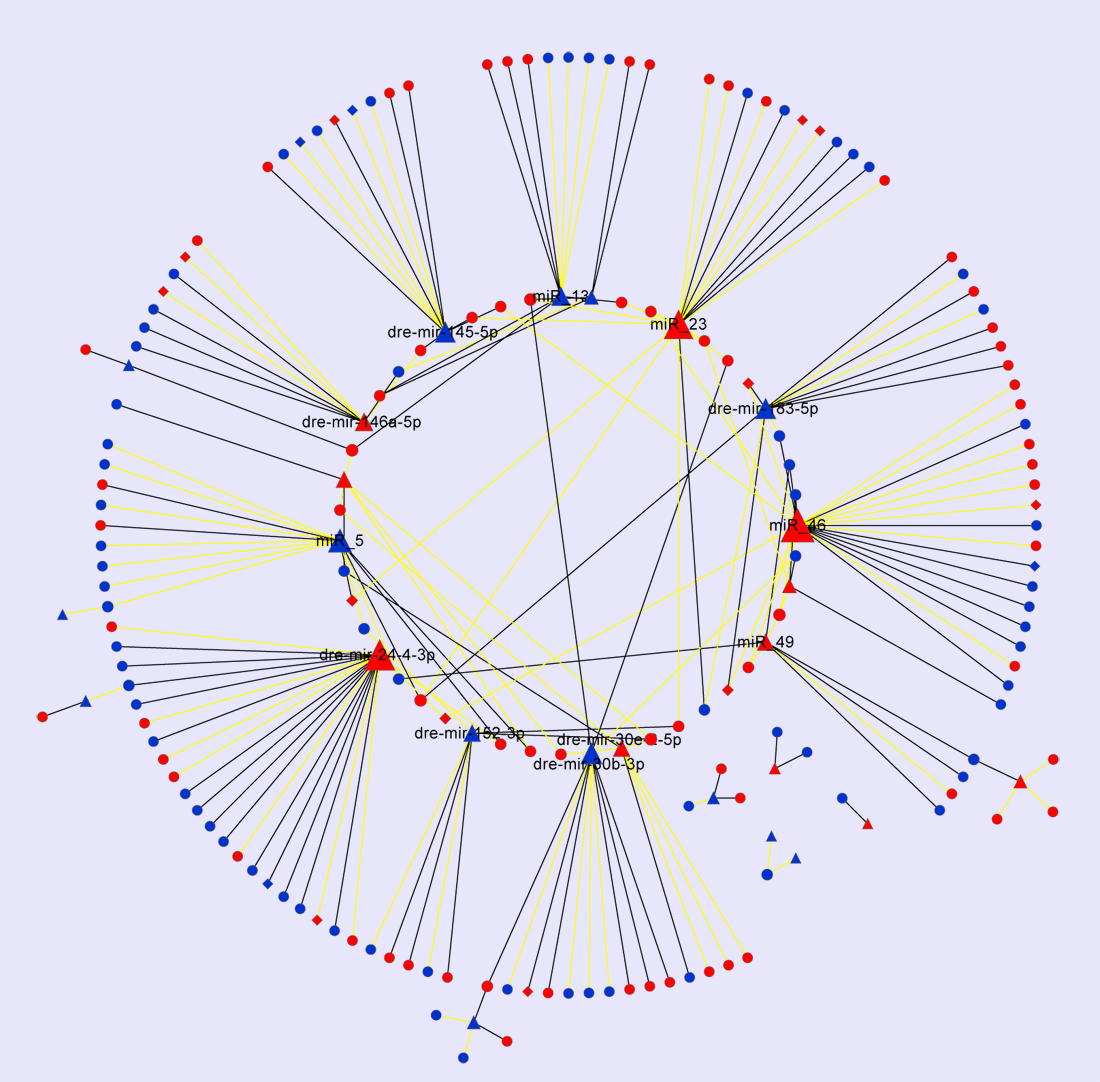

Supplement: Additional file 10 — Figure S4. Graphic illustration of the regulatory relationship between the differential miRNAs and mRNAs as predicted by miRanda. The style of this graph is the same as Figure 6. [file 1471-2164-12-605-S10.TIFF]
