# Supplementary material for: MicroRNA-mediated gene regulation plays a minor role in the transcriptomic plasticity of cold-acclimated Zebrafish brain tissue
Source: BMC Genomics. 2011 Dec 14;12:605. doi: 10.1186/1471-2164-12-605 (PMC3258298; doi:10.1186/1471-2164-12-605)
Supplement: Additional file 5 — RT-PCR verification of newly identified miRNAs. [file 1471-2164-12-605-S5.DOC]

**
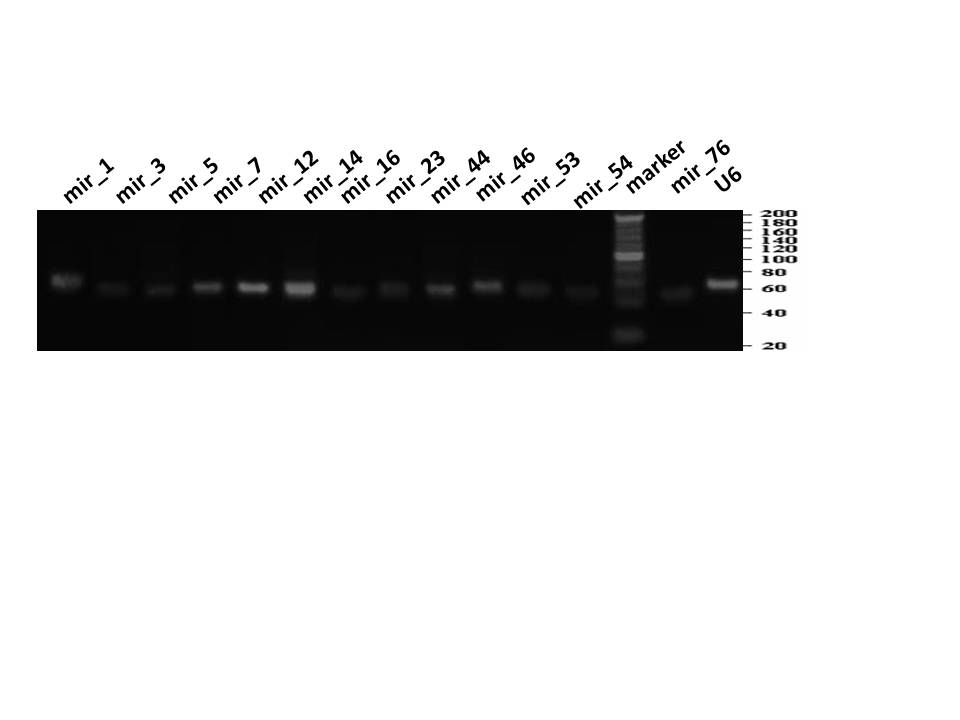
**

**RT-PCR verification of newly identified miRNAs.** Small RNAs (<200bp) were isolated from the total RNAs of zebrafish brain by mirVana^TM^ miRNA Isolation kit (Ambion，CA, USA), PolyA tail was added to the small RNAs using the Poly(A) Polymerase (PAP) enzyme and ATP. The RNAs were then reverse transcribed using a special oligo dT primer (5’-ATTCTAGAGGCCGAGGCGGCCGACATGT24VN-3’). PCR was performed 35 cycles at 95℃ for 30 seconds, 54℃ for 30 seconds and 72℃ for 45 seconds by using the selected 13 miRNA sequences (see the following table) as the upstream primer paired with the oligo dT primer as the downstream primer. The PCR products were run on a 4% agarose gel and photographed. In addition, the PCR products were also cloned into a TA cloning vector pMD18-T (Takara, CA, USA) and sequenced. All of the PCR products exhibited the correct miRNA-polyA-tail organization. These results indicate that the novel miRNAs we predicted were truly expressed as mature miRNAs in the Zebrafish brain. Note that the band labeled “U6” is 60 bp.

Thirteen miRNAs (out of the 399 miRNAs newly predicted) expressed at various levels were selected for verification. The following table lists their sequences and associated expression levels.

| **miRNA ID** | **MiR sequence** | **#cold-acclimated** | **#normal** |
| --- | --- | --- | --- |
| miR_1 | TACACGAGAACACCAGGACACA | 80614 | 26915 |
| miR_3 | ATGACTCAAACCCGAGGACTT | 1279 | 1618 |
| miR_5 | GCATTGGTGGTTCAGTGGGA | 698 | 1247 |
| miR_7 | TGACTCAAACCCGAGGACTCG | 879 | 850 |
| miR_12 | ATGACTCAAACCCGAAGACT | 134 | 198 |
| miR_14 | ACCTGTAACCATTGACTTCCAT | 308 | 170 |
| miR_16 | AAAGCGTACCAAACCGAA | 363 | 141 |
| miR_23 | AGCTGGTGTCCTGCAGAGTTT | 345 | 85 |
| miR_44 | ATCACTGAGCTGATGGAGAC | 85 | 33 |
| miR_46 | AAGAGAAGAGTGAGCGAGTGA | 149 | 32 |
| miR_53 | AACACGGCTAGTGACTGGTCAA | 55 | 27 |
| miR_54 | TGGAAGTCAATGGTAACCAGTT | 84 | 27 |
| miR_76 | ATGACTCAAACCCGATGACTT | 1 | 17 |
